# Long noncoding RNA ATB promotes ovarian cancer tumorigenesis by mediating histone H3 lysine 27 trimethylation through binding to EZH2

**DOI:** 10.1111/jcmm.15329

**Published:** 2020-12-18

**Authors:** Xue‐Juan Chen, Na An

**Affiliations:** ^1^ Department of Gynecology Shengli Oilfield Central Hospital Dongying Shandong China

**Keywords:** enhancer of zestehomolog 2, histone H3 Lys 27 trimethylation, lncRNA‐ATB, ovarian cancer, polycomb repressive complex 2

## Abstract

Ovarian cancer (OC) remains one of the most lethal gynecological malignancies. The unfavourable prognosis is mainly due to the lack of early‐stage diagnosis, drug resistance and recurrence. Therefore, it needs to investigate the mechanism of OC tumorigenesis and identify effective biomarkers for the clinical diagnosis. It is reported that long noncoding RNAs (lncRNAs) play important roles during the tumorigenesis of OC. Therefore, the present study aimed to study the role and clinical significance of LncRNAs ATB (lnc‐ATB) in the development and progression of OC. In our research, lnc‐ATB expression in OC tissues was elevated compared with adjacent normal tissues and high expression of lnc‐ATB was associated with poor outcomes of OC patients. The silencing of lnc‐ATB blocked cell proliferation, invasion and migration in SKOV3 and A2780 cells. RNA immunoprecipitation and RNA pull‐down results showed that lnc‐ATB positively regulated the expression of EZH2 via directly interacting with EZH2. Besides, the overexpression of EZH2 partly rescued lnc‐ATB silencing‐inducing inhibition of cell proliferation, invasion and migration. Chromatin immunoprecipitation assay results demonstrated that the silencing of lnc‐ATB reduced the occupancy of *caudal‐related homeobox protein 1*, *Forkhead box C1*, *Large tumour suppressor kinase 2*, *cadherin‐1* and *disabled homolog 2 interacting protein* promoters on EZH2 and H3K27me3. These data revealed the oncogenic of lnc‐ATB and provided a novel biomarker for OC diagnosis. Furthermore, these findings indicated the mechanism of lnc‐ATB functioning in the progression of OC, which provided a new target for OC therapy.

## 1 INTRODUCTION

Ovarian cancer is one of the leading causes of death and has become a severe public health problem.^1^ Although many patients respond to chemotherapy and surgical operation, the prognosis of OC patients remains unsatisfactory.^2^ Also, OC patients often had a poor outcome because of the lack of early‐stage diagnosis, rapid proliferation and metastasis.^3^ Therefore, it needs to study the underlying mechanisms of OC progression, explore novel biomarkers for early diagnosis and develop effective treatments for OC.

Long noncoding RNA (lncRNAs) are a new group of evolutionarily conserved RNA transcripts longer than 200 nucleotides in length with limited protein‐coding capacity.^4^ Convincing evidence elucidates the critical roles of lncRNAs in a large number of malignancies by repressing tumour suppressors or activating oncogenes.^4^ Long noncoding RNA activated by TGF‐β (lnc‐ATB) was identified to locate in chromosome 14 and is abnormally expressed in a variety of human malignant cancers, such as papillary thyroid cancer,^5^ hepatocellular carcinoma,^6^ gastric cancer^7^ and cervical cancer.^4^ High expression of lnc‐ATB promoted cell proliferation and metastasis and indicated poor prognosis in several cancers, such as non‐small cell lung cancer,^8^ osteosarcoma,^9^ breast cancer^10^ and renal cell carcinoma.^11^ However, the biological significance of lnc‐ATB and its potential role in OC remains to be documented. The molecular basis of lncRNA in performing their biological function is complex, including binding to RNA, DNA or protein.^12^ It is reported that Lnc‐ATB induced glioma malignancy by negatively regulating miR‐200a^13^ and promoted gastric cancer growth through a feedback loop of miR‐141‐3p/TGFβ2.^7^ Besides, lnc‐ATB participated in the progression of renal cell carcinoma by reducing p53 expression through the interaction with DNMT1.^14^ Lnc‐ATB was involved in autophagy by inducing Yes‐associated protein and up‐regulating ATG5 expression in HCC.^15^

Enhancer of zeste homolog 2 (EZH2) is a histone H3 lysine 27‐specific methyltransferase (H3K27me) of the polycomb repressive complex 2 (PRC2).^16^ EZH2 is highly expressed in various cancers, such as breast cancer^17^ and ovarian cancer.^18^, ^19^ High expression of EZH2 in OC promoted cell proliferation and correlated with a high proliferative index and tumour grade in OC.^20^ Therefore, EZH2 potentially serves as an effective therapeutic target. In the present study, we aimed to explore the clinical significance of lnc‐ATB and the mechanism of lnc‐ATB functioning in OC. Lnc‐ATB was highly expressed in OC tissues and cell lines, whereas silencing of lnc‐ATB blocked cell proliferation, invasion and migration in OC cell SKOV3 and A2780. RIP and RNA pull‐down results showed that lnc‐ATB directly bound to EZH2. In addition, overexpression of EZH2 rescued lnc‐ATB silencing‐inducing inhibition of proliferation, invasion and migration of SKOV3 and A2780 cells. ChIP assay results demonstrated that the silencing of lnc‐ATB reduced the occupancy of *caudal‐related homeobox protein 1* (*CDX1*), *Forkhead box C1* (*FOXC1*), *Large tumour suppressor kinase 2* (*LATS2*), *cadherin‐1* (*CDH1*) and *disabled homolog 2 interacting protein* (*DAB2IP*) promoters on EZH2 and H3K27me3. These findings revealed that lnc‐ATB exerted as an oncogene and provided the mechanism by which lnc‐ATB promotes the progression of ovarian cancer, which shed a new light for OC therapy.

## 2 MATERIALS AND METHODS

### 2.1 Patients and tissue samples

A total of 80 pairs of OC tissues and adjacent non‐tumour tissues were extracted from patients who had undergone surgical resections or biopsies at Shengli Oilfield Central Hospital. No patients received hormone therapy, chemotherapy or radiotherapy before the current study. All specimens were evaluated by at least two pathologists according to the WHO classification. Each patient signed an informed consent form. This study was approved by the Ethics Committee of Shengli Oilfield Central Hospital. Fresh tissue samples were immediately placed in a liquid nitrogen tank for follow‐up experiments.

### 2.2 Cell culture and transient transfection

The normal human ovarian epithelial cell (HOSE, BNCC340096), ovarian cancer cell lines SKOV3 (BNCC310551), A2780 (BNCC341157), IGROV1 (BNCC342341) and ES‐2 (BNCC100168) were purchased from BeNa Culture Collection (Beijing, China). Ovarian cell line OV2008 (CL1349) was obtained from the Institute of Biochemistry and Cell Biology of the Chinese Academy of Sciences (Shanghai, China). Cells were incubated with RPMI‐1640 medium containing 10% foetal bovine serum, 1% penicillin/streptomycin at 37°C in a humidified chamber supplemented with 5% CO_2_. Negative control (NC) and Lnc‐ATB siRNAs were synthesized by Sangon (Shanghai, China). The sequences against Lnc‐ATB and NC were as follows: si‐lnc‐ATB#1, 5'‐CUGUGUAGUUGUUUGUUAACU‐3'; si‐lnc‐ATB#2, 5'‐GCUGUGCAGUCUCAGGUUAGG‐3'; NC siRNA sense, 5'‑AGCAUGCAU GAGUACCCAGCC‑3'. The open reading frame of EZH2 was inserted into pcDNA3.1 (pcDNA3.1‐EZH2) for EZH2 overexpression. Cells were transfected with siRNAs or pcDNA3.1‐EZH2 by using RNAiMAX or Lipofectamine 3000 (Invitrogen, USA) according to the manufacturer's instruction. After transfection for the indicated time, the cells were harvested for further experiments.

### 2.3 Cell counting kit‐8 (CCK‐8) assay

Cells were seeded into 96‐well plates at the density of 3000 cells/well after the IncRNA‐ATB siRNA and NC siRNA transfection. After 0, 24, 48, 72 and 96 hours, 20 μL of CCK8 reagent was added into each well. After 2 hours at 37°C, the absorbance was measured at a wavelength of 490 nm on an automatic microplate reader (Dynex Technologies). Three independent experiments were repeated.

### 2.4 Colony formation assay

For the colony formation assay, 300 cells were seeded into 6‐well plates after the transfection of LncRNA‐ATB or NC siRNAs. After 10 d, cells were fixed with methanol for 10 minutes at room temperature and stained with 0.5% crystal violet solution (Beyotime Institute of Biotechnology) for 30 minutes at room temperature. Colonies were observed under an Olympus microscope, and the number of colonies was recorded.

### 2.5 Cell migration and invasion assays

Transwell chamber inserts (8.0 mm) were used for cell migration and invasion assays according to the manufacture's protocol. Briefly, the upper chamber was seeded with NC siRNA or LncRNA‐ATB siRNA‐transfected cells (1 × 10^4^) in 200 μL of serum‐free RPMI‐1640 medium, whereas, RPMI‐1640 containing 10% FBS was added to the lower chamber. After 36 hours, migrated or invasive cells were fixed with methanol and stained with 1% crystal violet for 20 minutes. The numbers of migrated or invasive cells were calculated from five random fields by using a light microscope (400×, Olympus). Experiments were carried out independently in triplicate. For cell invasion assay, the upper chamber was pre‐coated with Matrigel (Millipore).

### 2.6 Western blot

Proteins were extracted from cells using Radio‐Immunoprecipitation Assay buffer (RIPA, Beyotime Institute of Biotechnology), and the concentration of proteins was calculated by a BCA protein assay kit (Beyotime Institute of Biotechnology). An equal amount of proteins (20 μg/lane) was subjected to 12% SDS‐PAGE, followed by an electrophoretic transfer onto a polyvinylidene fluoride membrane. After being blocked with 5% non‐fat milk dissolved in Tris‐buffered saline containing 0.1% Tween‐20 (TBST) for 1 hour at room temperature, membranes were incubated with rabbit polyclonal antibodies against EZH2 (ab186006, 1:1,000, Abcam) or β‐actin (ab8227, 1:2,000, Abcam) overnight at 4°C. The next morning, membranes were washed with TBST three times and probed with horseradish peroxidase‐conjugated (HRP) goat anti‐rabbit IgG H & L secondary antibody (ab6721, 1:10,000, Abcam) for 1 hour at 37°C. After being washed with TBST, immunoreactive bands were developed by enhanced chemiluminescence (Beyotime Institute of Biotechnology) and the grey values of each blot were calculated by ImageJ version 1.50 (National Institutes of Health). The relative expression of the target protein was calculated by normalization to β‐actin.

### 2.7 Biotin‐labelled RNA pull‐down assay

Biotin‐labelled RNAs were synthesized using Biotin RNA labelling Mix (Roche) and T7 RNA polymerase (Promega), treated with RNase‐free DNase I (Promega) and purified with RNeasy Mini Kit (Qiangen). Next, 3 μg of biotin‐labelled RNAs was mixed with RNA structure buffer (10 mmol/L Tris, pH 7.0, 0.1 mol/L KCl and 10 mmol/L MgCl_2_) for 20 minutes at room temperature. Then, 10^7^ cells were lysed with 2 mL nuclear separation buffer and 6 mL H_2_O on ice for 20 minutes. After being centrifuged, the nuclear fraction was resuspended with 1 mL RIP buffer and homogenized 20 minutes. Streptavidin‐agarose beads (Sigma) were incubated with the supernatant for 1 hour and incubated with 5× loading buffer at 95°C for 5 minutes. The specificity of the RNA pull‐down was assessed using Western blot analysis.

### 2.8 Real‐time quantitative PCR (RT‐qPCR)

Total RNA from cells was isolated using Trizol reagent (Invitrogen) according to the manufacturer's protocol. The RNA concentration and quality were determined by A260/A280 by using a Nanodrop Spectrophotometer (IMPLEN GmbH). The first stranded cDNA was transcribed by using Super M‐MLV reverse transcriptase (BioTeke Corporation). RT‐qPCR was carried out by using Maxima SYBR Green/ROX qPCR Master Mix (Thermo Fisher Scientific) in an ABI 7500 Real‐Time PCR system (Applied Biosystems). PCR reaction conditions were as follows: 95°C for 50 seconds, followed by 40 cycles of 95°C for 15 seconds and 60°C for 45 seconds. The primers for RT‐qPCR were listed as follows: LncRNA‐ATB forward primer; 5′‐ggcaggtagaaaagtcggct‐3′, reverse primer: 5′‐tggaaagagtgggaaggatt‐3′; β‐actin forward primer: 5′‐cttagttgcgttacaccctttcttg‐3′, reverse primer: 5′‐ctgtcaccttcaccgttccagttt‐3′. The relative expression of target genes was normalized to *β‐actin* by the 2^−ΔΔCT^ method.^21^

### 2.9 RNA immunoprecipitation (RIP)

RNA immunoprecipitation was carried out by using the EZ‐Magna RIP RNA‐binding protein immunoprecipitation kit (Millipore, Billerica, MA, USA) according to the protocol. Cells were transfected with NC siRNA or LncRNA‐ATB siRNA and then lysed by RIP buffer. The collected supernatant was divided into two equal parts. A portion of the supernatant was analysed by Western blot analysis and RT‐qPCR to examine endogenous protein content. The other was treated with RIP buffer containing magnetic beads conjugated with anti‐EZH2 antibody or rabbit immunoglobulin G (IgG, Vector laboratories, Inc) at 4°C overnight. After instantaneous centrifugation, the supernatant was discarded. After six washes, the eluted samples were digested with 0.5 mg/mL proteinase K at 55°C for 30 minutes to detach the protein, and Trizol‐chloroform was employed to extract the immunoprecipitated RNA. Purified RNA was used for RT‐qPCR analysis.

### 2.10 Chromatin immunoprecipitation (ChIP)

ChIP was carried out by using EZ‐Magna ChIP TMA kits (Millipore) according to the manufacture's protocol. Cells were cross‐linked with 1% formaldehyde for 10 minutes and quenched with 125 mmol/L glycine for 5 minutes. Fixed cells were resuspended with cell lysis buffer (10 mmol/L Tris‐HCl, pH 7.5, 10 mmol/L NaCl, 0.5% NP‐40) with the mixture of protease inhibitors for 10 minutes on ice. After washes, the lysates were resuspended with sonication buffer and cracked with ultrasound to cut chromatin fragments into 200‐1000 bp. Protein A Agarose/Salmon Sperm DNA was added into the supernatant diluted with ChIP Dilution Buffer. After centrifugation, the supernatant was extracted and incubated with antibodies against EZH2, H3K27me3 (ab6002, Abcam), or IgG for immunoprecipitation overnight. DNA fragments in the immunoprecipitated complex were calculated by RT‐qPCR analysis. The input was used as the control and the relative signals in target groups were compared with the signal of the control IgG group.

### 2.11 Immunohistochemistry (IHC)

The sections of the tumour and adjacent non‐tumour tissues were fixed in 4% cold paraformaldehyde and cut into 3‐5 μm thickness. Sections were treated with 3% hydrogen peroxide in methanol at room temperature for 20 minutes for the blockage of the activity of endoperoxidase. After 3% BSA blockage, sections were incubated with rabbit polyclonal anti‐EZH2 antibody (ab186006, 1:500, Abcam) at 4°C overnight. The next morning, the sections were incubated with goat anti‐rabbit IgG H & L (HRP) secondary antibody (ab6721, 1:10,000, Abcam) for 1 hour at 37°C. After being washed with PBS, signals from five non‐overlapped high‐powered fields were developed with diaminobenzidine‐H_2_O_2_ solution and captured with an inverted microscope (Nikon, Japan). EZH2‐positive cells were stained in brown or dark nankeen.

### 2.12 Statistical analysis

Data were presented as mean ± standard deviation (SD). Each experiment was repeated at least three times. All statistical analysis was performed with SPSS 18.0 (IBM, SPSS). The comparison of the expression level of LncRNA‐ATB between OC and adjacent non‐tumour tissues was performed by using Student's *t* test. Kaplan‐Meier survival analysis was used to evaluate overall survival, and differences in survival between the curves were analysed with the two‐sided log‐rank test. Data among multiple groups were compared using one‐way analysis of variance. The Pearson correlation was used to analyse the relationship between Lnc‐ATB and EZH2. A value of *P* < .05 was considered statistically significant.

## 3 RESULTS

### 3.1 High expression of lnc‐ATB is correlated with poor outcome of OC patients

**FIGURE 1:**
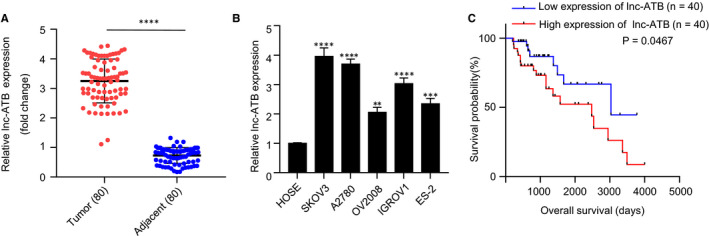
High expression of lnc‐ATB is correlated with poor outcome of OC patients. A, lnc‐ATB was highly expressed in OC tissues. RNA was isolated from 80 pairs of OC and adjacent non‐cancer tissues for RT‐qPCR analysis. **P < .01. B, lnc‐ATB was up‐regulated in OC cells. HOSE: normal human ovarian epithelial cell; OC cell lines: SKOV3, A2780, IGROV1, ES‐2 and OV2008. RNA was extracted from the cell lines and RT‐qPCR was analysed. **P < .01. C, High expression of lnc‐ATB was correlated with poor outcome of OC patients. Survival curves were plotted for low‐ and high‐expression groups using Kaplan‐Meier method with the log‐rank *t* test

To explore the potential function of lnc‐ATB in the progression of ovarian cancer, we determined lnc‐ATB expression levels in 80 pairs of ovarian tumour and adjacent non‐tumour tissues by RT‐qPCR. Compared with adjacent non‐tumour tissues, lnc‐ATB expression levels in OC tissues were significantly enhanced (*P* < .01, Figure 1A). Afterwards, we examined the expression of lnc‐ATB in normal human ovarian epithelial cells (HOSE) and five OC cell lines (SKOV3, A2780, OV2008, IGROV1 and ES‐2). Lnc‐ATB expression levels in SKOV3, A2780, OV2008, IGROV1 and ES‐2 cells were significantly higher than that in HOSE (*P* < .01, Figure 1B). Moreover, the levels of lnc‐ATB in SKOV3 and A2780 cells were highest. We chose SKOV3 and A2780 cells for further lnc‐ATB knockdown experiments. OC tissues were divided into low expression and high expression groups based on the median level of lnc‐ATB (Cut‐off). We then further assessed the relationship between lnc‐ATB expression and overall survival time of OC patients. Survival curves were plotted for low‐ and high‐expression groups by using Kaplan‐Meier method with the log‐rank *t* test. As shown in Figure 1C, overall survival probability with high lnc‐ATB expression was significantly lower than that of patients with low lnc‐ATB levels (*P* = .0467). These results showed that lnc‐ATB is highly expressed in OC tissues and cells, and the up‐regulation expression predicts poor outcome of OC patients.

### 3.2 Knockdown of lnc‐ATB blocks cell proliferation, migration, invasion in vitro

**FIGURE 2:**
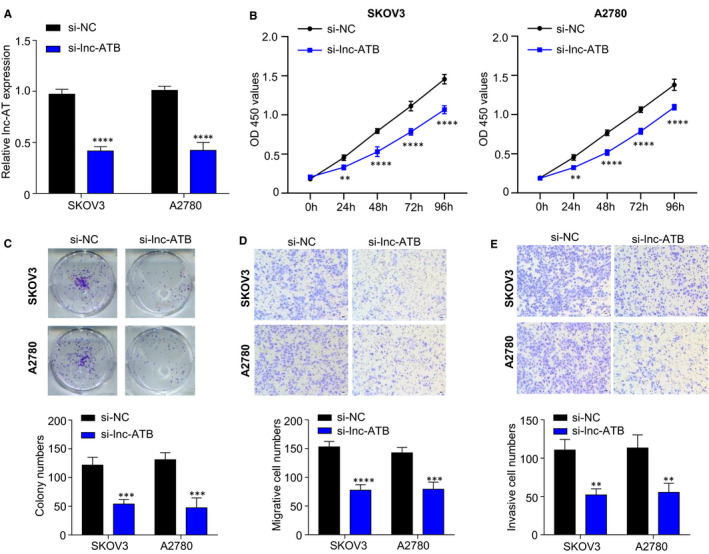
Silencing lnc‐ATB inhibits OC cell proliferation, migration, invasion in vitro. A, lnc‐ATB siRNAs (si‐lnc‐ATB #1 and si‐lnc‐ATB #2) transfection blocked the expression of lnc‐ATB in SKOV3 and A2780 cells analysed by RT‐qPCR. B, The blockage of lnc‐ATB suppressed the cell viability determined by CCK‐8 assay. C, The silencing of lnc‐ATB decreased the colony numbers examined by colony formation assay. D and E, The knockdown of lnc‐ATB decreased the migrated and invasive cell numbers determined by Transwell assay. SKOV3 and A2780 cells were transfected with lnc‐ATB siRNA or NC siRNA, respectively. After 48 h, RT‐qPCR, CCK‐8, colony formation and Transwell assays were carried out. **P < .01

To further identify the biological functions of lnc‐ATB in OC, we transfected SKOV3 and A2780 cells with synthesized NC or lnc‐ATB siRNAs and performed CCK‐8, colony formation, and Transwell assays. RT‐qPCR results showed that the transfection of lnc‐ATB siRNAs (si‐lnc‐ATB #1 or si‐lnc‐ATB #2) into SKOV3 and A2780 cells significantly inhibited the expression of lnc‐ATB compared with NC siRNA transfection in Figure 2A (*P* < .01), suggesting lnc‐ATB was successfully knocked down. As shown in Figure 2B, CCK‐8 assays results revealed that lnc‐ATB knockdown inhibited SKOV3 and A2780 cell viability compared with NC siRNA transfection from 24 to 96 hours in SKOV3 and A2780 cells. Furthermore, colony formation assay indicated that the silencing of lnc‐ATB significantly decreased colony numbers compared with NC siRNA‐transfected cells in Figure 2C. Transwell assays showed that the numbers of invaded and migrated cells were obviously attenuated in lnc‐ATB silencing group compared with NC group (*P* < .01, Figure 2D,E). These data showed that lnc‐ATB serves as an oncogene during OC biological process.

### 3.3 Lnc‐ATB directly binds to EZH2 and promotes the expression of EZH2

**FIGURE 3:**
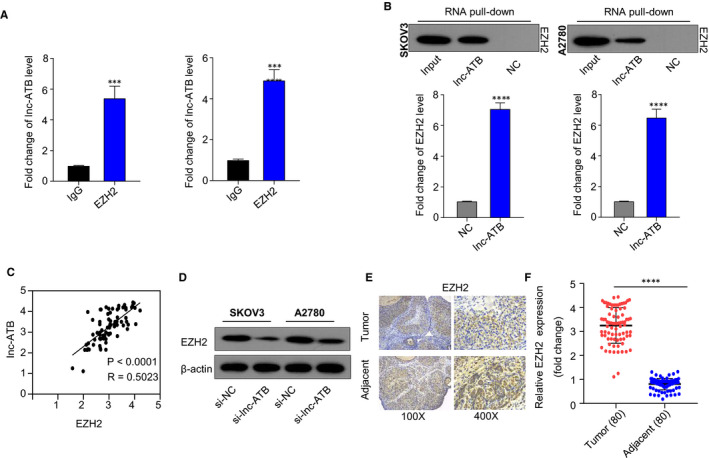
Lnc‐ATB directly binds to EZH2 and promotes the expression of EZH2. A, The enrichment level of lnc‐ATB in the EZH2 group was significantly higher than that in IgG group analysed RIP assay. B, lnc‐ATB directly bound to EZH2 determined by RNA pull‐down. C, EZH2 expression was positively associated with lnc‐ATB expression by Pearson analysis. D, The silencing of lnc‐ATB suppressed the protein levels of EZH2 in SKOV3 and A2780 cells. E, The signals of EZH2 in OC tissues were stronger than those in adjacent non‐cancer tissues determined by IHC. F, The mRNA levels of EZH2 in OC tissues were higher than those in adjacent non‐cancer tissues. **P < .01

To explore the detailed mechanism of Lnc‐ATB promoting OC development, we predicted the relative interaction probabilities between lnc‐ATB and RNA‐binding proteins online by using the RNA‐Protein Interaction Prediction database (http://pridb.gdcb.iastate.edu/RPISeq/) and found the relationship between lnc‐ATB and the sequence of EZH2. RIP assay results showed that the enrichment level of lnc‐ATB in the EZH2 antibody group was higher than that in control group, further verifying the binding relationship between EZH2 and lnc‐ATB (Figure 3A). RNA pull‐down results showed that biotin‐labeled lnc‐ATB pulled down EZH2 protein compared with biotin‐labeled NC group (Figure 3B). These data revealed that lnc‐ATB directly bound to EZH2. We performed Pearson's analysis and found that EZH2 expression was positively associated with lnc‐ATB expression in Figure 3C. In SKOV3 and A2780 cells, the silencing of lnc‐ATB significantly inhibited the protein levels of EZH2 (Figure 3D), confirming that lnc‐ATB positively regulated the expression of EZH2.

We further determined the relationship between lnc‐ATB and EZH2 based on 80 pairs of OC and adjacent non‐tumour tissues. In Figure 3D,E, OC tissues presented with higher expression of EZH2 compared with adjacent non‐tumour tissues. These data indicated that lnc‐ATB positively regulates the expression of EZH2 by directly binding to EZH2.

### 3.4 Ectopic expression of EZH2 rescues lnc‐ATB silencing‐inducing blockage of proliferation, invasion and migration of OC cells

**FIGURE 4:**
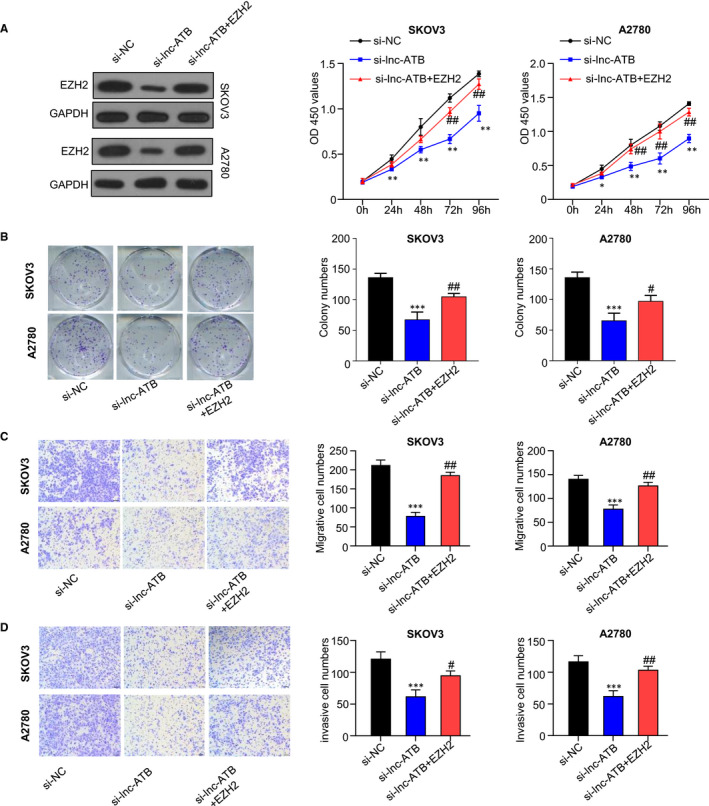
Overexpression of EZH2 rescues lnc‐ATB silencing‐inducing inhibition of proliferation, invasion and migration of OC cells. A, Western blots results showed that EZH2 was successfully overexpressed. B, Overexpression of EZH2 partly rescued lnc‐ATB knockdown induced blockage of cell viability determined by CCK‐8 assay. C, Overexpression of EZH2 partly increased the colony cell number reduced by lnc‐ATB silencing analysed by colony formation assay. D and E, EZH2 partly increased the migrated and invasive cell numbers decreased by lnc‐ATB silencing. SKOV3 and A2780 cells were randomly divided into three groups: si‐NC, si‐lnc‐ATB and si‐lnc‐ATB + EZH2. si‐lnc‐ATB and si‐lnc‐ATB + EZH2 groups were transfected with lnc‐ATB siRNA. si‐NC group was transfected with NC siRNA. After 48 h, cells in si‐lnc‐ATB + EZH2 group were transfected with pcDNA3.1‐EZH2. After 48 h, proteins were extracted for western blot. CCK‐8, colony formation and Transwell assays were performed. Compared with si‐NC, **P < .01; Compared with si‐lnc‐ATB group, ^#^ *P* < .05, ^##^ *P* < .01

We further examined whether the overexpression of EZH2 can rescue the silencing of lnc‐ATB‐induced blockage of cell proliferation, invasion and migration. As shown in Figure 4A, the transfection of pcDNA3.1‐EZH2 enhanced the protein levels of EZH2, suggesting EZH2 was overexpressed successfully in cells. In Figure 4B,C, the silencing of lnc‐ATB decreased cell viability. However, the overexpression of EZH2 partly rescued the decrease in cell viability. Similarly, colony formation and Transwell assays indicated that the overexpression of EZH2 partly rescued the knockdown of lnc‐ATB induced decline of colony numbers and the invasive and migrated cell numbers (Figure 4D,E). These data showed that the overexpression of EZH2 partly rescues lnc‐ATB knockdown‐inducing inhibition of proliferation, invasion and migration of OC cells.

### 3.5 Lnc‐ATB knockdown significantly increases the expression of a cohort of EZH2 target genes

**FIGURE 5:**
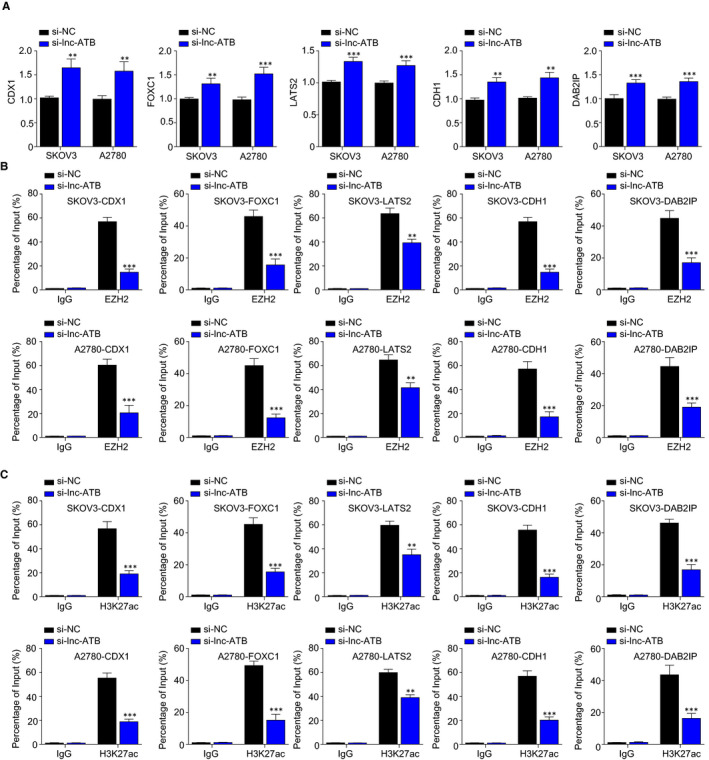
Lnc‐ATB knockdown significantly increases the expression of a cohort of EZH2 target genes. A, The silencing of lnc‐ATB promoted the expression of *CDX1*, *FOXC1*, *LATS2*, *CDH1* and *DAB2IP* in SKOV3 and A2780 cells. Cells were transfected with lnc‐ATB siRNA or NC siRNA, respectively. After 48 h, RNA was extracted for RT‐qPCR. B, The knockdown of lnc‐ATB decreased the enrichment of EZH2 in the *CDX1*, *FOXC1*, *LATS2*, *CDH1* and *DAB2IP* promoter regions determined by ChIP. C, The knockdown of lnc‐ATB reduced the occupancy of *CDX1*, *FOXC1*, *LATS2*, *CDH1* and *DAB2IP* promoters on H3K27me3. Cells were transfected with lnc‐ATB siRNA or NC siRNA, respectively. ChIP was performed with antibodies against EZH2, H3K27me3 or IgG. The relative signals were normalized to input and subsequently compared with the signal of IgG group. **P < .01

Previous reports showed that EZH2 targets a cascade of tumour suppressors, such as CDX1, FOXC1, LATS2, CDH1 and DAB2IP. We hypothesized that lnc‐ATB promoted the development of OC via suppressing the above targets of EZH2. In Figure 5A, the silencing of lnc‐ATB significantly released the expression of *CDX1*, *FOXC1*, *LATS2*, *CDH1* and *DAB2IP*, suggesting the negative relationship between lnc‐ATB and the above genes. In Figure 5B, ChIP detection indicated that inhibition of lnc‐ATB reduced the enrichment of EZH2 in the *CDX1*, *FOXC1*, *LATS2*, *CDH1* and *DAB2IP* promoter regions. In addition, the ChIP assay demonstrated that the knockdown of lnc‐ATB reduced the occupancy of *CDX1*, *FOXC1*, *LATS2*, *CDH1* and *DAB2IP* promoters on H3K27me3. These data showed that the silencing of lnc‐ATB decreased histone trimethylation of *CDX1*, *FOXC1*, *LATS2*, *CDH1* and *DAB2IP* promoters through recruiting EZH2 and promoted the expression of CDX1, FOXC1, LATS2, CDH1 and DAB2IP.

## 4 DISCUSSION

Increasing evidence reported that lnc‐ATB promoted the progression of various cancers, such as breast cancer,^22^ prostate carcinoma ^23^ and colon cancer.^24^ Thus, lnc‐ATB is predicted as a potential prognostic marker and therapeutic target of human cancers.^25^ In our present study, we found that lnc‐ATB exerted as an oncogene in OC and revealed that lnc‐ATB induces the development of OC via directly interacting with EZH2. These findings provided a new biomarker for OC diagnosis and a new target for OC therapy.

In our study, lnc‐ATB was highly expressed in OC tissues and cell lines, and the silencing of lnc‐ATB blocked cell proliferation, invasion and migration, suggesting that lnc‐ATB serves as an oncogene in OC. These data were in agreement with the previous study that up‐regulation of lnc‐ATB enhances proliferation, migration and invasion in papillary thyroid carcinoma cell ^26^ and lung cancer,^27^ which provided new evidence about the function of lnc‐ATB in human cancers. Our results indicated that lnc‐ATB is a promising diagnostic marker for OC. A cascade of reports showed that the mechanism of lnc‐ATB involving in the progression of human cancers was complex. In HCC, lnc‐ATB promoted cell invasion via TGF‐β/miR‐200s/ZEB signalling pathway.^28^ Lnc‐ATB regulated the growth and metastasis of cholangiocarcinoma ^29^ and colorectal cancer ^30^ via miR‐200c. In cervical cancer, lnc‐ATB promotes proliferation and invasion by regulating the miR‐144/ITGA6 axis.^4^ In lung cancer, lnc‐ATB promoted proliferation and metastasis via down‐regulating miR‐494.^27^ However, in our study, we found that lnc‐ATB could directly bind to EZH2 and lnc‐ATB is positively correlated with the expression of EZH2. In addition, the overexpression of EZH2 partly rescued the inhibition of cell proliferation, invasion and migration induced by lnc‐ATB. These data revealed lnc‐ATB promoted the development of OC via binding to EZH2, which shed new light on the mechanism of lnc‐ATB functioning in human cancers.

PRC2 is a transcription‐repressive complex consisted of three components: EZH2, embryonic ectoderm development (EED) and suppressor of zeste 12 (SUZ12).^31^ It is reported that PRC2 plays a crucial role in epigenetic regulation of normal development and malignancy.^32^ The catalytic subunit EZH2 belongs to the polycomb group protein family and can trimethylate lysine 27 on histone 3 (H3K27) to mediate gene transcription repression by facilitating chromatin compaction.^17^ Epigenetic alteration often leads to dysregulated gene expression. LATS2 is one of the Hippo members,^33^ and low LATS2 was associated with better survival in OC.^34^ E‐cadherin is one of epithelial‐mesenchymal transformation markers and increased E‐cadherin inhibits cell invasion of OC.^35^ It is reported that EZH2 directly interact with LATS2 and E‐cadherin promoter regions and activate H3K27 trimethylation modification in non‐small cell lung cancer ^36^ and gastric cancer.^37^ A scaffold protein DAB2IP exerts as a tumour suppressor by regulating cell proliferation, and it is epigenetically down‐regulated in a large number of tumours through EZH2.^38^ In our study, we found that the silencing of lnc‐ATB significantly up‐regulated the expression of LATS2, CDH1 and DAB2IP, and decreased the binding of EZH2 and H3K27me3 across LATS2, CDH1 and DAB2IP promoters in OC cell lines. These results indicated that lnc‐ATB interacted with EZH2 occupancy and epigenetically modulated the expression of LATS2, CDH1 and DAB2IP. We also found that EZH2 could directly target the promoters of CDX1, FOXC1 and induce the methylation. We further demonstrated that the down‐regulated transcriptions of CDX1 and FOXC1 were mediated by the H3K27me3. Therefore, we considered CDX1 and FOXC1 are targets of EZH2 in ovarian cancer. Although the anti‐tumour roles of CDX1 and FOXC1 have been reported,^39^, ^40^ more evidence about their function in repressing ovarian cancer should be provided in future.

**FIGURE 6:**
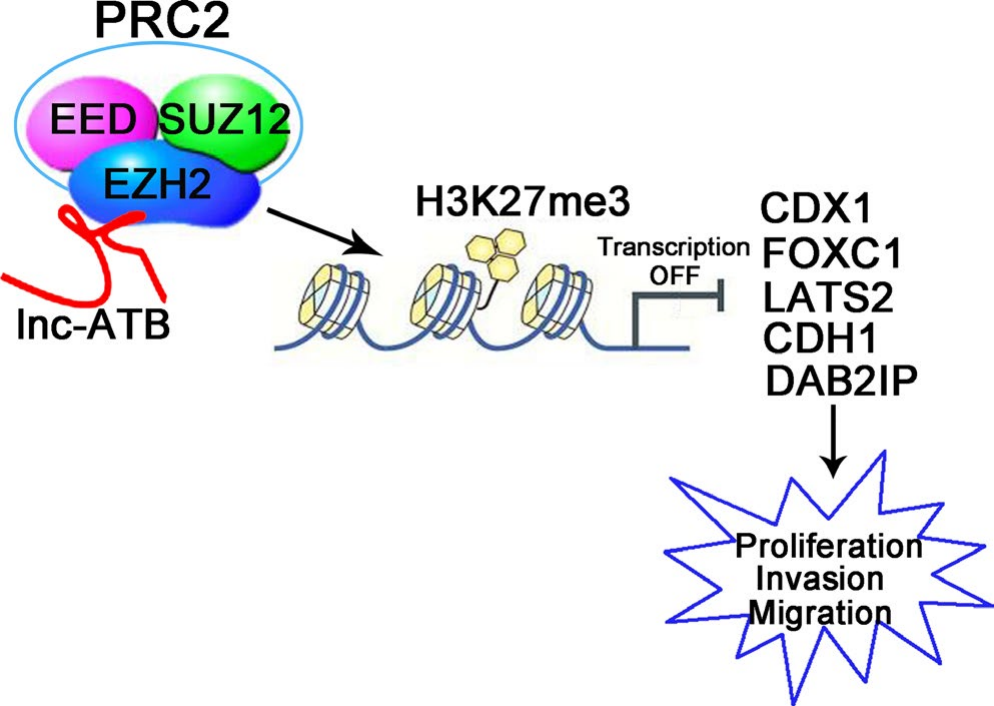
Schematic diagram demonstrating how lnc‐ATB was involved in OC progression. The PRC2 complex consists of EZH2, EED, and SUZ12. lnc‐ATB directly binds to EZH2 and mediates its accumulation at the promoter region of *CDX1*, *FOXC1*, *LATS2*, *CDH1* and *DAB2IP* genes, leading to the trimethylation of H3K27 and the inhibition of *CDX1*, *FOXC1*, *LATS2*, *CDH1* and *DAB2IP* expression

In summary, lnc‐ATB is overexpressed in OC, and high expression of lnc‐ATB is correlated with the progression of OC. Besides, lnc‐ATB directly binds to EZH2 and mediates its accumulation at the promoter region of *CDX1*, *FOXC1*, *LATS2*, *CDH1* and *DAB2IP* genes, leading to the trimethylation of H3K27 and the inhibition of tumour suppressors *CDX1*, *FOXC1*, *LATS2*, *CDH1* and *DAB2IP* expression. That is to say, lnc‐ATB promotes OC progression by the blockage of *CDX1*, *FOXC1*, *LATS2*, *CDH1* and *DAB2IP* via EZH2‐mediated epigenetic silencing (Figure 6). These findings showed that lnc‐ATB may represent a novel biomarker for OC diagnosis, prognosis and therapy.

## CONFLICT OF INTEREST

All authors declare no conflicts of interest in this work.

## Data Availability

Data available on request from the authors.
